# Prognostic Significance of Immune Checkpoint Markers in Prognosis of Grade 3 Endometrioid Carcinoma

**DOI:** 10.3390/medicina62020327

**Published:** 2026-02-06

**Authors:** Emine Kilic Bagir, Umran Kucukgoz Gulec, Semra Paydas, Ahmet Baris Guzel, Mehmet Ali Vardar, Gulsah Seydaoglu, Derya Gumurdulu

**Affiliations:** 1 Department of Pathology, Faculty of Medicine, Cukurova University, 01330 Adana, Türkiye; 2 Department of Obstetrics and Gynecology, Faculty of Medicine, Cukurova University, 01330 Adana, Türkiye; 3 Department of Medical Oncology, Faculty of Medicine, Cukurova University, 01330 Adana, Türkiye; 4 Department of Biostatistics, Faculty of Medicine, Cukurova University, 01330 Adana, Türkiye

**Keywords:** PD-1, PD-L1, prognosis, immunotherapy, high-grade endometrioid carcinoma

## Abstract

*Background and Objectives*: Uterine FIGO grade 3 endometrioid carcinoma (EC) is an uncommon but aggressive subtype of endometrial cancer with limited biomarker data to guide prognosis and management. This study aimed to evaluate the prognostic significance of programmed death-1 (PD-1) and programmed death-ligand 1 (PD-L1) expression in tumor tissue (TT) and tumor microenvironment (TME). *Materials and Methods*: We retrospectively analyzed tumor samples from 53 patients with FIGO grade 3 EC. Immunohistochemistry was performed to assess PD-1 and PD-L1 expression in TT and TME. Clinicopathological data including age, stage, lymph node invasion (LNI), lymphovascular space invasion (LVSI), depth of myometrial invasion (MI), adjuvant therapy, and survival outcomes were collected. Survival analyses were conducted using Kaplan–Meier and Cox proportional hazards models. *Results*: PD-1 expression was identified in 34% of TT and 41.5% of TME, while PD-L1 was expressed in 22.6% of TT and 34% of TME. Except for PD-1 in TME, positive expression of these immune checkpoint molecules correlated with significantly shorter survival (log-rank *p* < 0.05) outcomes. In univariate analysis, PD-1 and PD-L1 expression in TT, deep MI, LNI and LVSI were associated with adverse outcomes. Multivariate analysis confirmed PD-1 and PD-L1 positivity in TT as independent prognostic factors (PD-1: HR 3.2, 95% CI 1.4–7.0; PD-L1: HR 3.3, 95% CI 1.4–7.8). Patients with concurrent PD-1 and PD-L1 expression in TT showed the poorest overall survival, suggesting a cumulative negative effect. *Conclusions*: PD-1 and PD-L1 expression in tumor tissue are independent predictors of poor prognosis in FIGO grade 3 EC. These findings support their role as clinically relevant biomarkers and potential therapeutic targets. Incorporating checkpoint evaluation into routine pathological assessment could improve prognostic accuracy and guide treatment strategies, particularly in high-risk patients who might benefit from immunotherapy approaches.

## 1. Introduction

Endometrioid carcinoma (EC) of the uterus is the most common subtype of endometrial carcinoma. According to the FIGO classification, grade 3 EC is diagnosed when the tumor contains more than 50% solid growth without squamous differentiation or when a grade 2 EC demonstrates marked cytologic atypia, excluding glandular variants of serous carcinoma [1]. High-grade endometrial carcinomas (HGEC), which include FIGO grade 3 EC, serous, and clear cell carcinomas, are associated with poor clinical outcomes. However, grade 3 EC appears to have an intermediate prognosis between uterine serous or clear cell carcinoma and low-grade (grade 1–2) EC, as shown in population-based analyses involving more than 200,000 patients [2]. These findings suggest the need for distinct treatment strategies tailored to the biology of HGEC.

In recent years, the tumor immune microenvironment has gained increasing attention in endometrial cancer research. Immune checkpoint pathways, particularly the programmed death-1 (PD-1) and programmed death-ligand 1 (PD-L1) axis, play a critical role in tumor immune evasion [3]. Aberrant PD-1/PD-L1 signaling has been shown to negatively influence prognosis in several malignancies, and checkpoint inhibitors targeting this pathway have become a cornerstone of cancer immunotherapy. PD-1 is expressed in malignant tumors, and anti-PD-1 antibody therapy has been approved by the FDA for various cancers [4]. However, identifying reliable biomarkers to predict which patients will benefit from such therapies remain a major clinical challenge.

Endometrial carcinoma is a heterogeneous and biologically complex disease. Its molecular and histopathological diversity strongly influence prognosis and therapeutic response, underscoring the importance of biology-driven treatment approaches [5,6]. PD-1 and PD-L1 expression have been reported to serve as both prognostic and predictive biomarkers for checkpoint blockade therapies (CBTs) in uterine cancers.

The aim of this study was to investigate PD-1 and PD-L1 expression in tumor tissue (TT) and tumor microenvironment (TME) of patients with FIGO grade 3 EC, and to evaluate their potential prognostic impact.

## 2. Materials and Methods

This retrospective study included 53 patients diagnosed with high-grade endometrial carcinoma (HGEC) who underwent surgery and follow-up at our university hospital, and whose paraffin blocks were available from the pathology archive. We received approval from the local ethical committee. Tissue samples that were suitable in terms of tumor and microenvironment from patient materials were selected for immunohistochemical analysis. Demographic variables such as age, menopausal status, parity, medical and cancer history, and presenting symptoms were recorded. Clinicopathological data included depth of myometrial invasion (MI), International Federation of Gynecology and Obstetrics (FIGO 2009) stage [7], lymphovascular space invasion (LVSI), lymph node involvement (LNI), adjuvant therapies, and survival outcomes. The primary surgical procedures consisted of laparotomic or laparoscopic total hysterectomy with bilateral salpingo-oophorectomy (TH + BSO), combined with pelvic and para-aortic lymphadenectomy, with or without omentectomy. Postoperative adjuvant therapy was administered based on individual risk profiles, with chemotherapy for systemic control and radiotherapy for local control. Follow-up evaluations were performed every 3 months during the first year and every 6 months thereafter for up to 5 years. Clinical assessment included gynecological examination, transvaginal ultrasonography, and thoracic and abdominopelvic computed tomography as indicated. Overall survival (OS) was defined as the interval, in months, from surgery or diagnosis to death or the last follow-up.

### 2.1. Immunohistochemical Staining

Formalin-fixed, paraffin-embedded tumor tissues were sectioned at 5 µm and stained using monoclonal antibodies against PD-1 (MRQ-22, Ventana, Tucson, AZ, USA) and PD-L1 (CD274/PD-L1, Acris, Heidelberg, Germany). Staining was performed on the BenchMark XT platform with enzymatic digestion and the iView Blue Detection Kit (Ventana). In tumor tissue (TT), only complete or incomplete membranous staining of tumor cells was considered positive, whereas in the tumor microenvironment (TME), both membranous and cytoplasmic staining in tumor-infiltrating lymphocytes. Staining intensity was graded as 0 (negative), 1+ (weak), 2+ (moderate), or 3+ (strong). A cut-off of ≥1% positively stained cells was used for both tumor tissue (TT) and tumor microenvironment (TME). The TME includes immune cells, vascular structure and lymphatics, fibroblasts, and pericytes. The assessment of PD-1 and PD-L1 expression in the microenvironment was performed on tumor-infiltrating lymphocytes (TILs). In this cohort, no specimens demonstrated 2+ or 3+ staining; therefore, cases were divided into two groups: positive and negative. Tonsil tissue served as a positive control.

### 2.2. Statistical Analysis

Data were analyzed using IBM SPSS Statistics v20.0 (IBM Corp., Armonk, NY, USA). Normality was assessed with the Shapiro–Wilk test. Descriptive statistics were expressed as mean ± standard deviation (SD). To compare categorical data, Chi-square and Fisher’s exact tests were used. A two-tailed *p* value < 0.05 was accepted as statistically significant. We performed the Kaplan–Meier method to evaluate the association between PD-1 and PD-L1 expressions and survival times. The Cox proportional-hazards model was applied to analyze the significance of multiple variables. Data were analyzed using IBM SPSS Statistics v20.0 (IBM Corp., Armonk, NY, USA).

## 3. Results

Fifty-three cases with FIGO grade 3 EC were assessed for PD-1 and PD-L1 expressions in TT and TME. The mean age was 59.0 ± 10.2 years (range: 32–72). Abnormal uterine bleeding was the most common presenting symptom (88.4%), and nearly half of the patients had comorbidities (%46.8). Demographic and clinical features were shown in Table 1. Early-stage disease (stage I–II) was present in 58.5% of the cohort, while deep myometrial invasion (>50%) was observed in 69.8%. LVSI was identified in 75% and lymph node invasion (LNI) in 37.7% of patients. Adjuvant chemotherapy and radiotherapy were administered in 45.3% and 79.2% of cases, respectively (Table 2).

**Figure 1:**
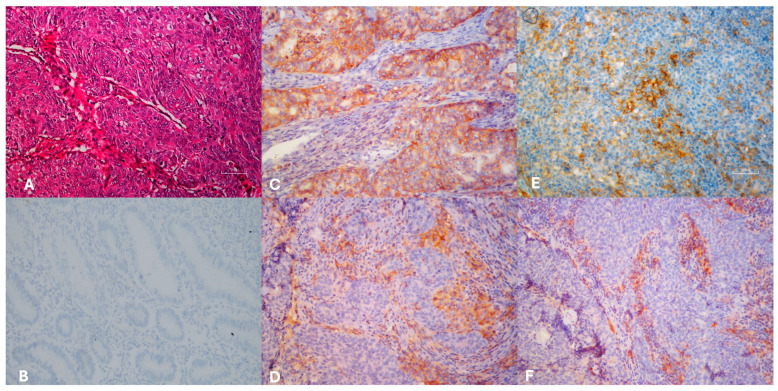
Hematoxylin eosin sections of endometrioid carcinoma FİGO grade 3 ((**A**), H&E×100). Immunohistochemically normal endometrium negative staining ((**B**), IHC×200), PD-L1 ((**C**), IHC×200) and PD-1 ((**E**), IHC×400) positivity of the tumor cells, PD-L1 ((**D**), IHC×200) and PD-1 ((**F**), IHC×200), positivity of the microenvironment.

**Figure 2:**
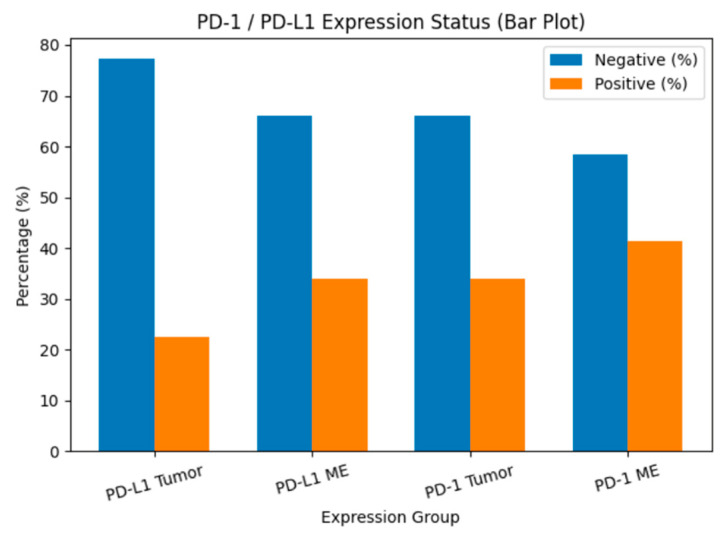
PD-1 and PD-L1 expression rates in tumor tissue and tumor microenvironment. Bar chart showing the percentage distribution of PD-L1 and PD-1 expression status in tumor cells (TT) and tumor microenvironment (TME). Blue bars represent cases without expression, while orange bars represent cases with positive expression.

**Figure 3:**
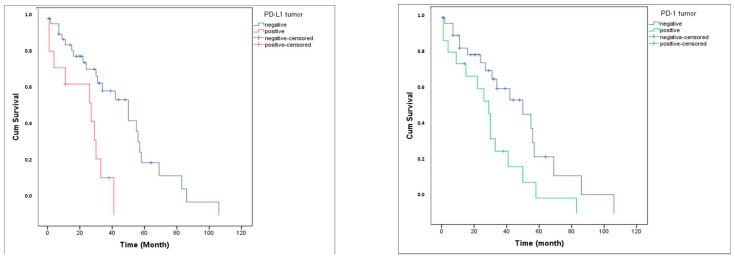
Overall survival curve according to PD-L1 and PD-1 expression.

PD-1 expression was detected in 34% of tumor tissues (TT) and 41.5% of tumor microenvironments (TME), whereas PD-L1 was expressed in 22.6% of TT and 34% of TME (Figure 1). The distribution of PD-1 and PD-L1 expression rates is summarized in Figure 2. Representative normal endometrium samples showed negative staining for PD-1 and PD-L1, as illustrated in Figure 1. Survival analysis demonstrated that PD-1 and PD-L1 expression in TT, as well as PD-L1 expression in TME, were significantly associated with reduced overall survival (OS), while PD-1 in TME showed no prognostic impact. Median OS was 30 months in PD-1 positive versus 55 months in PD-1 negative cases (*p* = 0.025), and 27 versus 50 months for PD-L1 positive and negative patients, respectively (*p* = 0.001). Kaplan–Meier survival curves illustrating overall survival differences according to PD-1 and PD-L1 expression are presented in Figure 3. Univariate analysis identified deep myometrial invasion (*p* = 0.010), LVSI (*p* = 0.030), LNI (*p* = 0.036), and PD-1/PD-L1 expression as significant prognostic factors (Table 3). Importantly, patients with concurrent PD-1 and PD-L1 positivity in TT experienced the poorest survival outcomes, suggesting a cumulative adverse effect of dual checkpoint expression. Multivariate Cox regression confirmed PD-1 and PD-L1 expression in TT as independent predictors of poor prognosis. PD-1 positivity conferred a 3.2-fold increased risk of mortality (HR 3.2; 95% CI 1.4–7), while PD-L1 positivity was associated with a 3.3-fold increased risk (HR 3.3; 95% CI 1.4–7.8). In addition, lymph node invasion (LNI) remained an independent adverse prognostic factor in multivariate analysis, showing a significantly increased risk of mortality in both models (Model 1: HR 3.2, 95% CI 1.1–9.5, *p* = 0.034; Model 2: HR 4.6, 95% CI 1.4–14.4, *p* = 0.010). Although LVSI and deep myometrial invasion (MI) were significant in univariate analysis, they did not remain independent predictors after adjustment in multivariate models. All univariate and multivariate survival analyses are summarized in Table 3 and Table 4.

In summary, PD-1 and PD-L1 expression in tumor tissue, but not in the microenvironment, emerged as independent adverse prognostic markers in FIGO grade 3 EC. The combined expression of both checkpoints identified a subgroup with particularly poor survival, highlighting their potential clinical relevance for prognostic stratification and therapeutic decision-making.

## 4. Discussion

Our findings support that PD-1 and PD-L1 expression in tumor tissue (TT) are independent prognostic factors and are associated with significantly worse overall survival in patients with FIGO grade 3 endometrioid carcinoma (EC). Although PD-1/PD-L1 positivity was present in a minority of cases, it identified a high-risk subgroup with significantly worse survival outcomes. This apparent paradox underscores that low prevalence of PD-1/PD-L1 expression does not preclude prognostic significance; rather, marker positivity delineates a biologically aggressive subset of tumors associated with significantly worse survival outcomes. Notably, lymph node invasion (LNI) also emerged as an independent predictor of poor prognosis in multivariate models. This finding aligns with established clinicopathological evidence that nodal involvement reflects systemic tumor spread and is strongly associated with worse survival outcomes in endometrial carcinoma. Importantly, the persistence of PD-1/PD-L1 tumor tissue expression as independent predictors even after adjustment for nodal status underscores their potential value for risk stratification beyond conventional prognostic factors. This finding is consistent with emerging literature that emphasizes the negative prognostic implications of immune checkpoint molecule expression, particularly in high-grade endometrial tumors [8,9,10,11,12].

Grade 3 EC represents a biologically unique entity, situated between low-grade endometrioid carcinoma and serous or clear cell histologies. Despite its relative rarity, it carries a considerable risk of recurrence and mortality yet remains underrepresented in biomarker-focused studies. Our exclusive focus on this subset offers a clinically relevant contribution to the existing knowledge base. By evaluating PD-1 and PD-L1 expression not only in tumor cells but also within the tumor microenvironment (TME), we aimed to highlight spatial immune evasion patterns and enrich the current understanding of immune modulation in these tumors.

Interestingly, PD-1 and PD-L1 expression in the TME did not show independent prognostic significance, a finding that contrasts with reports from other malignancies and even some subtypes of endometrial cancer. This discrepancy may reflect histologic or molecular heterogeneity, or alternatively, it could suggest that tumor-intrinsic checkpoint expression plays a more dominant role in clinical outcomes for grade 3 EC [13,14].

Previous studies have shown that PD-L1 expression is more frequent in tumors with microsatellite instability (MSI) or POLE mutations, and that these molecular subgroups often exhibit increased tumor-infiltrating lymphocytes (TILs) and mutational burden [15,16,17]. Furthermore, the ProMisE classifier has stratified ECs into four biologically distinct groups (POLE-mut, MMR-d, p53-abn, p53-wt) with differing prognosis and therapeutic vulnerabilities [18,19]. Grade 3 tumors are disproportionately represented in the p53-abn and MMR-d subgroups, both known for heightened immune checkpoint expression and varying immunotherapy response profiles. In our study, however, comprehensive molecular profiling (e.g., POLE mutation status, MMR protein expression, or p53 immunoreactivity) could not be performed due to retrospective design and institutional limitations. These tests, while clinically informative, were not part of routine pathological practice during the study period, and additional tissue or funding constraints prevented retrospective analysis.

Nevertheless, the clear and consistent association between tumor tissue PD-1/PD-L1 expression and poor survival across both univariate and multivariate analyses indicates that these markers may serve as accessible surrogate indicators of aggressive tumor biology, even in the absence of detailed molecular data. Our findings complement earlier work by Kim et al. and Pasanen et al., who similarly identified a link between PD-L1 expression and reduced survival in high-grade EC [9,20]. More recently, Fu et al. demonstrated in a large meta-analysis that PD-L1 positivity is significantly associated with poorer progression-free survival in endometrial cancers, particularly in high-grade and deeply invasive tumors [21]. In contrast, Proppe et al. showed that the prognostic value of PD-1/PD-L1 expression is strongly influenced by the applied scoring system, with PD-1 immune cell and combined positive scores correlating with improved disease-free survival, whereas PD-L1 expression alone was not consistently prognostic [22,23]. These contrasting findings highlight both the biological complexity of checkpoint expression and the urgent need for standardized scoring methodologies.

From a clinical standpoint, routine immunohistochemical evaluation of PD-1 and PD-L1 could represent a feasible and cost-effective tool for prognostic stratification, especially in settings where molecular testing is not widely accessible. Furthermore, such markers may help identify patients with particularly high-risk disease who could be prioritized for immunotherapy trials, especially as checkpoint inhibitors continue to expand their regulatory approval in the treatment of mismatch repair-deficient and advanced endometrial cancers [4,8].

We acknowledge the limitations inherent to our study. Its retrospective, single-center design and modest cohort size may limit generalizability. In addition, the modest cohort size may have limited the statistical power of multivariable analyses, and larger multicenter cohorts are needed to further validate our results. Furthermore, paired adjacent non-tumorous endometrial tissue was not consistently available across all cases; therefore, systematic quantitative comparisons between tumor tissue (TT), tumor microenvironment (TME), and normal/adjacent tissue could not be performed, although representative normal endometrium staining was included for reference. Moreover, the absence of additional immune marker analysis, such as CD8+ T cell density, or characterization of Treg and macrophage subpopulations, restricts our ability to fully capture the complexity of the TME. Despite these constraints, the internal consistency of our results, the narrow focus on a high-risk tumor group, and the dual-compartment evaluation (TT and TME) of checkpoint molecules enhance the reliability and clinical relevance of our findings. Future studies integrating external transcriptomic/genomic datasets (e.g., GEPIA, TIMER, and cBioPortal) and molecular subgroup stratification may further validate and expand the clinical significance of PD-1/PD-L1 expression in high-grade endometrial carcinoma.

## 5. Conclusions

PD-1 and PD-L1 expression in tumor tissue (TT) are adverse prognostic indicators in FIGO grade 3 endometrioid carcinoma, together with lymph node invasion (LNI), which also remained independently associated with poor survival in multivariate analysis. While comprehensive molecular and immune profiling were not feasible in our setting, our data supports the potential role of immunohistochemical assessment of these checkpoints as a practical surrogate marker to identify patients at higher risk. Future multicenter, prospective studies incorporating molecular data are essential to validate these findings and refine biomarker-guided prognostic stratification and individualized management approaches for high-grade endometrial carcinoma.

## Figures and Tables

**Table 1:** Demographic features of the patients.

		n	(%)
Age Groups	<55	14	(26.4)
	55–64	23	(43.4)
	>64	16	(30.2)
Parity	Yes	35	(66.0)
Menopause	Yes	42	(79.2)
Medical history	Cancer	2	(4.3)
	Comorbidity	22	(46.8)
Presenting symptom	Abnormally uterine bleeding	38	(88.4)
	Bloating	2	(4.7)
	Pain	3	(7.0)

**Table 2:** Pathological and clinical features and evaluation of their effects on prognosis.

				Prognosis				
		Total		Ex		Alive		
		n	%	n	%	n	%	*p*
Stage	1 + 2	31	(58.5)	12	(63.2)	19	(55.9)	
	3 + 4	22	(41.5)	7	(36.8)	15	(44.1)	0.606
LND	None	17	(32.1)	7	(36.8)	10	(29.4)	
	PLND	6	(11.3)	2	(10.5)	4	(11.8)	
	PPALND	30	(56.6)	10	(52.6)	20	(58.8)	0.857
Optimal cytoreduction	No	5	(9.4)	3	(37.5)	2	(10.5)	
	Yes	22	(41.5)	5	(62.5)	17	(89.5)	0.099
	Unknown	26	(49.1)					
MI	<50%	16	(30.2)	7	(36.8)	9	(26.5)	
	≥50	37	(69.8)	12	(63.2)	25	(73.5)	0.430
LNI	Negative	33	(62.3)	14	(73.7)	19	(55.9)	
	Positive	20	(37.7)	5	(26.3)	15	(44.1)	0.200
LVSI	Negative	13	(25.0)	5	(27.8)	8	(23.5)	
	Positive	39	(75.0)	13	(72.2)	26	(76.5)	0.736
PD-L1 tumor	Negative	41	(77.4)	17	(89.5)	24	(70.6)	
	Positive	12	(22.6)	2	(10.5)	10	(29.4)	0.115
PD-L1 ME	Negative	35	(66.0)	15	(78.9)	20	(58.8)	
	Positive	18	(34.0)	4	(21.1)	14	(41.2)	0.138
PD-1 tumor	Negative	35	(66.0)	17	(89.5)	18	(52.9)	
	Positive	18	(34.0)	2	(10.5)	16	(47.1)	**0.** **007**
PD-1 ME	Negative	31	(58.5)	13	(68.4)	18	(52.9)	
	Positive	22	(41.5)	6	(31.6)	16	(47.1)	0.273
Radiotherapy	No	7	(13.2)	4	(22.2)	3	(9.7)	
	Yes	42	(79.2)	14	(77.8)	28	(90.3)	0.226
	Unknown	4	(7.5)					
Chemotherapy	No	25	(47.2)	9	(50.0)	16	(51.6)	
	Yes	24	(45.3)	9	(50.0)	15	(48.4)	0.913
	Unknown	4	(7.5)					
Status	Alive	19	(35.8)					
	Ex	34	(64.2)					

LND: lymph node dissection; PLND: pelvic lymph node dissection; PPALND: pelvic and para-aortic lymph node dissection; LNI: lymph node invasion, LVSI: lymphovascular space invasion, MI: myometrial invasion; ME: microenviroment.

**Table 3:** The overall and disease-free survival rates according to univariate survival analyses.

		Overall Survival (Month)			DFS Survival (Month)		
		Mean	Median	*p* *	Mean	Median	*p* *
Stage	1 + 2	49.9	50.0		41.4	41.0	
	3 + 4	34.3	27.0	0.057	24.5	20,0	**0.020**
MI	<50%	64.4	58.0		46.8	50.0	
	≥50	34.4	30.0	**0.010**	28.9	27.0	0.076
LNI	Negative	50.4	50.0		39.6	34.0	
	Positive	33.0	26.0	**0.036**	25.6	22.0	0.063
LVSI	Negative	62.6	55.0		49.0	50.0	
	Positive	34.7	31.0	**0.030**	29.2	28.0	0.077
PD-L1 tumor	Negative	49.1	50.0		38.3	31.0	
	Positive	23.0	27.0	**0.001**	20.3	26.0	**0.014**
PD-L1 ME	Negative	50.3	50.0		42.0	34.0	
	Positive	27.6	27.0	**0.015**	19.7	12.0	**0.003**
PD-1 tumor	Negative	50.2	55.0		38.5	31.0	
	Positive	31.2	30.0	**0.025**	26.9	26.0	0.123
PD-1 ME	Negative	47.5	50.0		40.2	34.0	
	Positive	36.4	30.0	0.155	25.1	22.0	**0.041**
		42.8	41.0	34.0	33.9	29.0	

* Log-rank test. LNI: lymph node invasion, LVSI: lymphovascular space invasion, MI: myometrial invasion, ME: microenviroment.

**Table 4:** Results of two different model for multivariate Cox analysis contributing to prognosis.

	*p*	HR	95.0% CI for HR	
			Lower	Upper
Model 1				
Age	0.229	1.0	0.9	1.0
Stage	0.783	1.8	0.0	104.5
LNI	0.034	3.2	1.1	9.5
MI	0.775	1.8	0.0	110.2
PD-L1 tumor	0.007	3.3	1.4	7.8
Model 2				
Age	0.511	1.0	0.9	1.0
Stage	0.783	1.7	0.0	74.1
LNI	0.010	4.6	1.4	14.4
MI	0.730	2.0	0.0	88.0
PD-1tumor	0.005	3.2	1.4	7.0

LNI: Lymph node invasion, MI: myometrial invasion.

## Data Availability

Data is contained within the article.
